# Exploring the prognostic impact and biological functions of mutant-like *TP53*-related genes in acute myeloid leukemia

**DOI:** 10.1016/j.htct.2026.106455

**Published:** 2026-04-14

**Authors:** Letícia Machado Favery Bertoline, Juan Luiz Coelho-Silva, Keli Lima, Eduardo Magalhães Rego, João Agostinho Machado-Neto

**Affiliations:** a Department of Pharmacology, Institute of Biomedical Sciences, University of São Paulo (USP), São Paulo, Brazil; b Laboratory of Medical Research in Pathogenesis and Targeted Therapy in Onco-Immuno-Hematology (LIM-31), Department of Clinical Medicine, Division of Hematology, School of Medicine, University of São Paulo, São Paulo, Brazil

To the editor

Acute myeloid leukemia (AML) is an aggressive hematologic malignancy characterized by the clonal expansion of myeloid progenitor cells resulting from genetic and epigenetic alterations in hematopoietic stem or progenitor cells. These mutations disrupt normal differentiation and lead to the accumulation of immature myeloid blasts in the bone marrow, peripheral blood, and in other tissues, impairing normal hematopoiesis [1,2]. According to the 2022 World Health Organization (WHO) classification, AML is categorized into two major groups: AML with defined genetic abnormalities, which includes entities characterized by recurrent gene mutations or chromosomal rearrangements, and AML defined by differentiation, which is classified based on the predominant lineage and maturation stage of the leukemic blasts [3]. In parallel, the International Consensus Classification (ICC) proposed an alternative approach, creating new categories: AML with myelodysplasia-related cytogenetic abnormalities, AML with myelodysplasia-related gene mutations, and AML with mutated *TP53* [4]. Mutations in the *TP53* gene are strongly associated with an unfavorable prognosis in AML and represent a challenge in current therapeutic management [5, 6, 7]. In the study conducted by Lee et al. [8], using machine learning-based analysis of gene expression, a specific genetic signature was identified in patients with AML harboring wild-type *TP53*, whose transcriptional profile closely mimics that of *TP53*-mutated AML. These patients, referred to as *TP53* mutant-like, also exhibit adverse clinical outcomes comparable to those observed in cases with *TP53* mutations. Given these findings, a detailed characterization of this molecular signature is essential to improve prognostic stratification and enable the selection of more appropriate therapeutic strategies. Such an approach could significantly enhance treatment response and overall quality of life for these patients. This study aims to explore the prognostic potential of *TP53* mutant-like-related genes and to develop a prognostic score based on the expression of key genes, capable of defining risk groups with greater precision than traditional stratification systems that rely solely on molecular and cytogenetic risk factors.

Patients with non-M3 AML who had received potentially curative therapy, had wild-type *TP53* status, and had RNA-seq data available in the TCGA (*n* = 113) and Beat AML (*n* = 197) cohorts were included in the analysis [9,10]. Dichotomization of gene expression was performed based on receiver operating characteristic (ROC) curve analysis and the concordance index (C-index). Overall survival (OS) was defined as the time, in months, from diagnosis to the date of last follow-up or death. Survival analyses were performed using Kaplan-Meier curves and compared with the log-rank test and/or Cox proportional hazards regression. For continuous variables, Student’s *t*-test, ANOVA, Kruskal-Wallis, or Mann-Whitney tests were applied as appropriate, while categorical variables were analyzed using the chi-square test or Fisher’s exact test in GraphPad Prism 8 (GraphPad Software, Inc.), Stata Statistic/Data Analysis 14.1 (Stata Corp., College Station, TX, USA), or SPSS Statistics for Windows, version 21.0 (SPSS, Chicago, IL, USA). All transcripts from the TCGA AML RNA-seq dataset were pre-ranked according to their differential expression by comparing samples stratified by the 3-gene *TP53* mutant-like score (low, intermediate, and high). This analysis was performed using the normalized quantile method and the Limma-Voom package implemented in Galaxy (https://usegalaxy.org/). A heatmap was generated using ClusterVis (https://biit.cs.ut.ee/clustvis), while volcano and correlation plots were produced with SRplot (https://www.bioinformatics.com.cn/srplot). Gene set enrichment analysis (GSEA) was performed with GSEA v.4.0.15, using the Hallmark gene sets curated by MSigDB. Enrichment scores (ES) were calculated based on the Kolmogorov-Smirnov statistic and tested for significance using 1000 permutations. The scores were normalized (NES) to account for the size of each gene set. All differentially expressed genes (p-value <0.05) obtained from Galaxy were used for Gene Ontology (GO) enrichment analysis through the ShinyGO v0.77 database (http://bioinformatics.sdstate.edu/go/), and the top upregulated and downregulated GO biological processes were illustrated. Statistical significance was defined as p-value <0.05 and/or false discovery rate (FDR) <0.25.

From the analysis, four of the 25 genes initially associated with the *TP53* mutant-like profile showed a significant impact on overall survival (OS) in both cohorts, with three genes, *PEAR1, UGCG*, and *NUDT13*, exhibiting consistent effects in the same direction (p-value <0.05) (Figure 1A and B). Based on the hazard ratio (HR) values corresponding to each gene, a prognostic score, termed the “3-gene *TP53* mutant-like score”, was developed. This score stratified patients into three distinct risk categories: low, intermediate, and high risk. In the Beat AML cohort, a significant reclassification of patients was observed when comparing the conventional European LeukemiaNet (ELN) 2022 risk classification with the new 3-gene *TP53* mutant-like score-based classification (Figure 1D). The intermediate ELN group was predominantly redistributed into the intermediate and high-risk categories of the new system. Moreover, patients previously classified as favorable risk were almost evenly redistributed across the three new categories (Figure 1D). Compared with the ELN 2022 classification, the 3-gene *TP53* mutant-like score identified a greater proportion of patients within the intermediate and high-risk groups (Figure 1D). A similar redistribution pattern was observed in the TCGA cohort when comparing the new score with traditional molecular and cytogenetic risk stratification systems (Figure 1E). In AML patients stratified according to the ELN 2022 criteria, the 3-gene *TP53* mutant-like score provided additional prognostic value within the favorable-risk group (p-value = 0.0002; Supplementary Figure 1). When patients were stratified based on *FLT3* and *NPM1* mutation status, a trend toward reduced overall survival was observed in those with a high 3-gene *TP53* mutant-like score, reaching statistical significance in the *NPM1* mutated Beat AML cohort (p-value = 0.006; Supplementary Figure 2). Details of the included AML patients, as well as the associations between the 3-gene *TP53* mutant-like score and clinical or laboratory features, are presented in Supplementary Tables 1 and 2. In summary, the high-score group showed higher leukocyte counts and was associated with poorer risk stratification categories. Although the score correlated with parameters typically linked to adverse prognosis in AML [11], multivariate analyses indicated that the 3-gene score is an independent prognostic factor (Supplementary Tables 3 and 4) in both analyzed cohorts. A limitation of this study is the heterogeneity of the original datasets in terms of AML subtypes, disease stage, and treatment regimens, which may influence clinical outcomes, as well as the reliance on RNA-seq data that require independent validation by quantitative polymerase chain reaction-based methodologies to support potential clinical application.

**Fig. 1:**
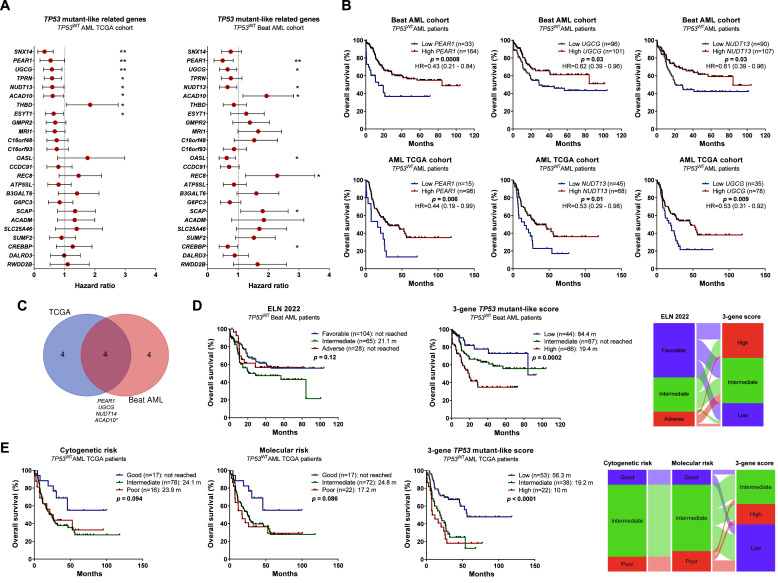
Prognostic impact of the 3-gene TP53 mutant-like score in AML. (A) Hazard ratio (HR), 95% confidence interval (95% CI), and p-values (Log-rank test) for overall survival (OS) associated with the 25 genes previously related to mutant-like TP53 in the TCGA and Beat AML cohorts. **(B)** Kaplan–Meier curves showing OS for AML patients stratified into low and high expression groups for each gene, using the ROC curve-defined cutoff. HR, 95% CI, and p-values (Log-rank test) are shown. **(C)** Venn diagram showing the overlap of genes significantly impacting prognosis in AML across both cohorts. The genes *PEAR1, UGCG*, and *NUDT4* were consistently associated with prognosis in the same direction in both datasets. **(D)** Kaplan–Meier curves depicting OS for Beat AML patients stratified according to the ELN 2022 classification and the 3-gene TP53 mutant-like score. HR, 95% CI, and p-values (Log-rank test) are shown. The schematic illustrates patient reclassification according to the ELN 2022 and 3-gene TP53 mutant-like score. **(E)** Kaplan–Meier curves showing OS for Beat AML patients stratified according to cytogenetic risk, molecular risk, and the 3-gene TP53 mutant-like score. HR, 95% CI, and p-values (Log-rank test) are shown. The schematic highlights patient redistribution when applying the 3-gene TP53 mutant-like score compared with conventional risk classifications.

From a biological perspective, *PEAR1* participates in intracellular signaling and cell-cell interactions, *UGCG* regulates glycosphingolipid biosynthesis and membrane-associated signaling, and *NUDT13* contributes to mitochondrial nucleotide metabolism and oxidative stress control, collectively linking signal transduction, membrane biology, and cellular metabolic homeostasis.

Subsequently, using functional genomics, we sought to evaluate the biological and molecular processes differentially regulated according to the risk stratification defined by the 3-gene *TP53* mutant-like score. The most pronounced differences in gene expression signatures were observed between the high and low 3-gene *TP53* mutant-like score groups (Figure 2A and B). Genes upregulated in the high- versus low-score group were predominantly associated with enhanced mitochondrial metabolism, oxidative phosphorylation, ATP production, and purine biosynthesis, whereas those downregulated in the high- versus low-score group were mainly related to mitosis, cell cycle progression, and response to external stimuli (Figure 2C). GSEA analyses further corroborate these findings (Figure 2D). These findings are consistent with recent studies showing that increased mitochondrial metabolism and biomass production are associated with chemoresistance and poor prognosis in AML [12, 13, 14]. Finally, when exploring potential associations between the 3-gene *TP53* mutant-like score and specific mutational signatures, we identified an enrichment of *FLT3* and *NPM1* mutations within the intermediate- and high-risk groups defined by the new score (Figure 2E). Overall, the establishment of this score underscores its potential as a robust tool for refining prognostic stratification in AML and may contribute to the development of more personalized therapeutic approaches.

**Fig. 2:**
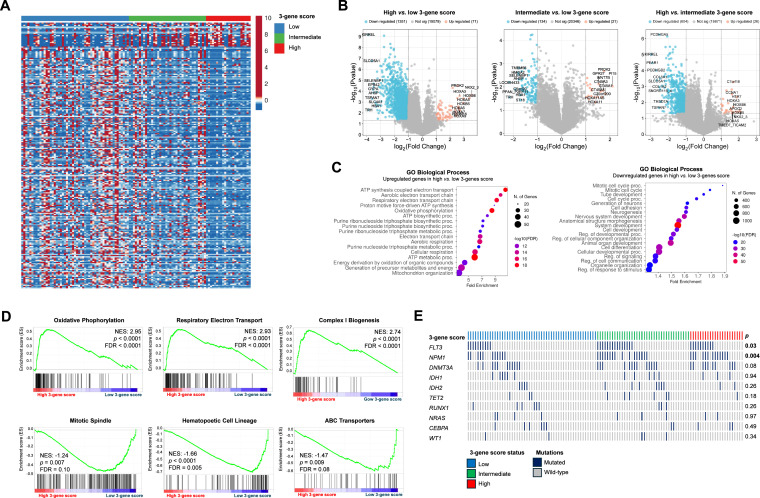
Functional genomic characterization of the 3-gene TP53 mutant-like score in AML. (A) Heatmap summarizing the expression patterns of the three genes. Color intensity represents the z-score normalized within each row. **(B)** Volcano plots illustrate the magnitude (x-axis) and statistical significance (y-axis) of differential gene expression across comparisons of high vs. low, high vs. intermediate, and intermediate vs. low groups according to the 3-gene TP53 mutant-like score. **(C)** Biological processes enriched among upregulated and downregulated genes in the high vs. low 3-gene TP53 mutant-like score groups. **(D)** Gene set enrichment analysis (GSEA) in the TCGA AML cohort showing that higher expression levels of the 3-gene TP53 mutant-like score are significantly enriched for pathways related to oxidative phosphorylation, respiratory electron transport, and complex I biogenesis, while being depleted for mitotic spindle organization, hematopoietic cell lineage, and ABC transporter activity. NES, FDR, and *p*-values are indicated. **(E)** Analysis of mutational enrichment demonstrating the distribution of AML-relevant mutated genes across groups defined by the 3-gene TP53 mutant-like score.

In summary, the present study proposes a model that enables more accurate therapeutic decision-making, offering some patients, previously classified as low risk by conventional systems, a new therapeutic perspective and a potential opportunity for remission. The findings expand current evidence on the clinical relevance of the wild-type *TP53* transcriptional signature in AML, underscoring the importance of adopting more refined risk stratification strategies. The proposed 3-gene *TP53* mutant-like score demonstrates strong potential to overcome the limitations of existing prognostic models, while also providing insights into the underlying biological mechanisms that may inform the development of novel targeted therapies aimed at improving outcomes for patients with AML.

## Conflicts of interest

The authors declare no competing interests.

## Appendix Supplementary materials

Image, application 1

Image, application 2

Image, application 3

Image, application 4
