# Supplementary material for: Exploring the prognostic impact and biological functions of mutant-like TP53-related genes in acute myeloid leukemia
Source: Hematol Transfus Cell Ther. 2026 Apr 14;48(3):106455. doi: 10.1016/j.htct.2026.106455 (PMC13092737; doi:10.1016/j.htct.2026.106455)
Supplement: Supplementary file 1 [file mmc1.pdf]

Supplementary Figures

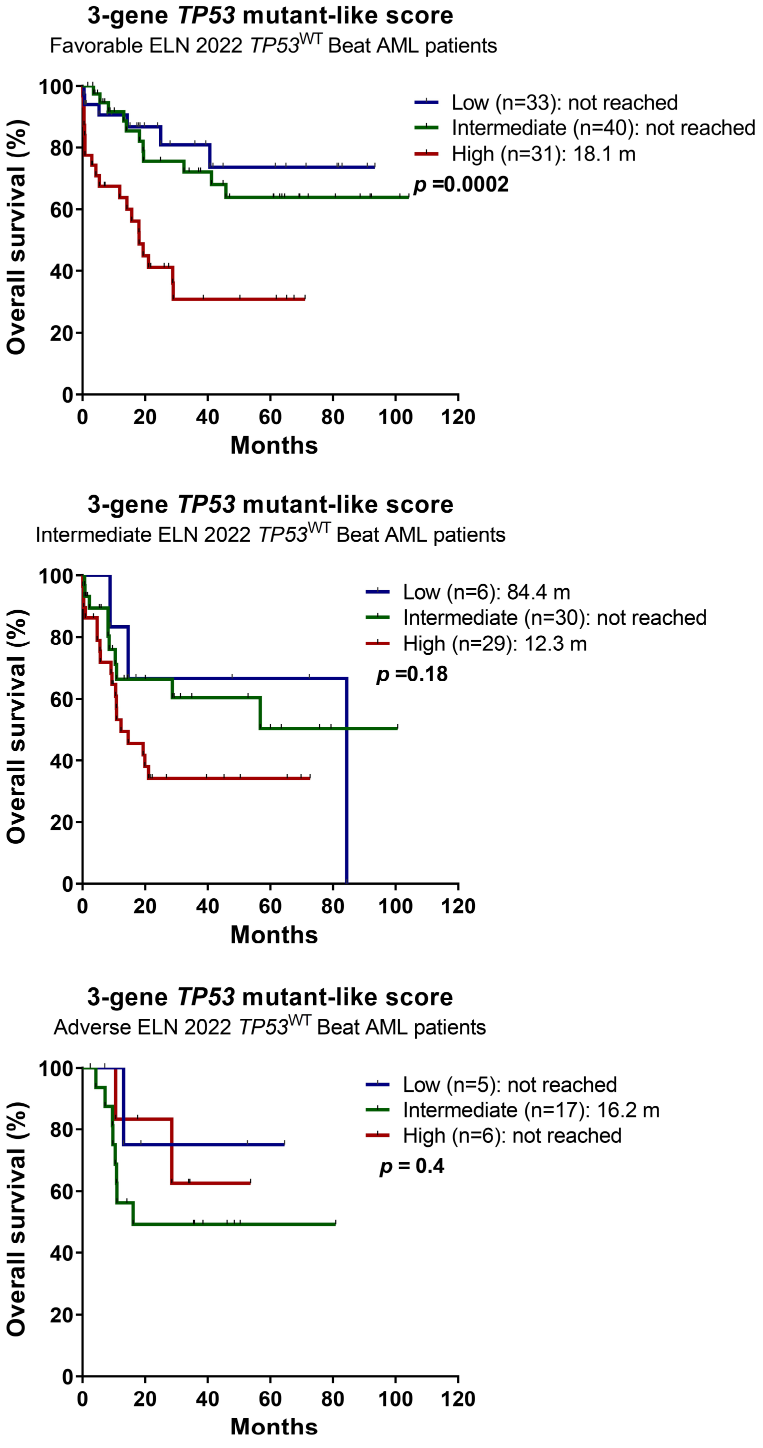

Supplementary Figure 1. Impact of the 3-gene TP53 mutant-like score across ELN 2022 risk categories in AML. Hazard ratios (HR), 95% confidence intervals (95% CI), and log-rank *p*-values for overall survival (OS) associated with the 3-gene TP53 mutant-like score in the Beat AML cohort, stratified according to ELN 2022 risk groups (favorable, intermediate, and adverse).

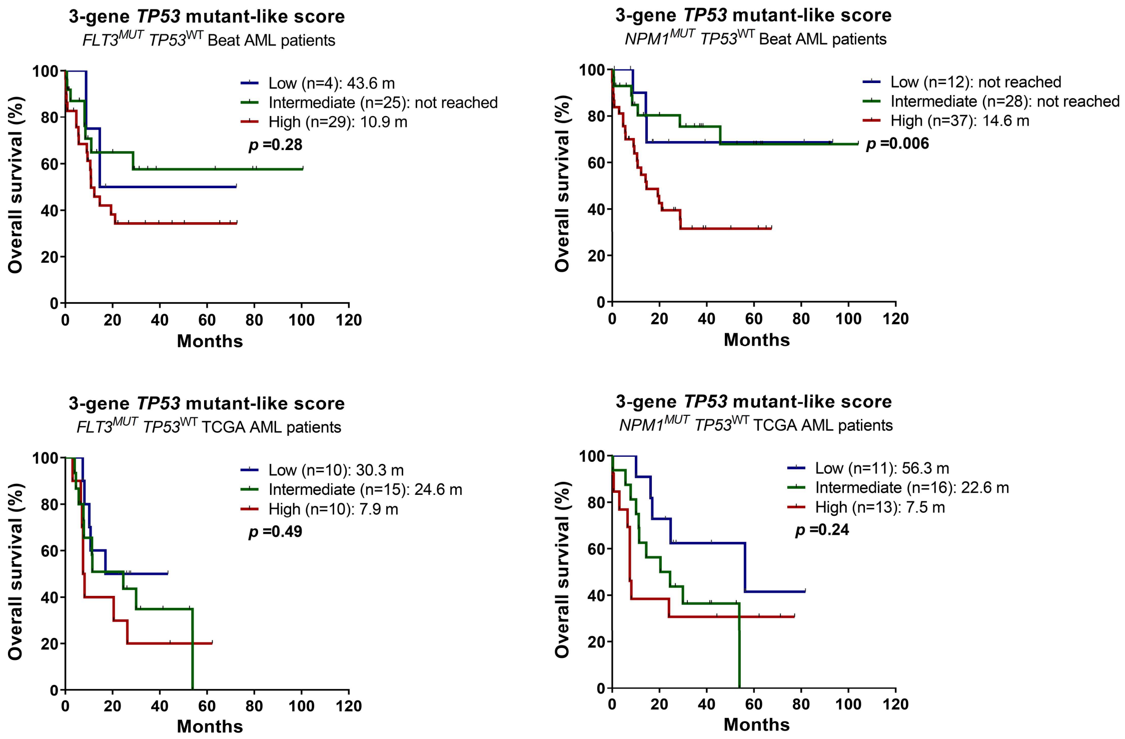

Supplementary Figure 2. Impact of the 3-gene TP53 mutant-like score in FLT3- and NPM1-mutated AML. Hazard ratios (HRs), 95% confidence intervals (95% CIs), and log-rank *p*-values for overall survival (OS) associated with the 3-gene TP53 mutant-like score in FLT3- and NPM1-mutated AML patients from the Beat AML and TCGA cohorts.
