# Supplementary material for: Exploring the prognostic impact and biological functions of mutant-like TP53-related genes in acute myeloid leukemia
Source: Hematol Transfus Cell Ther. 2026 Apr 14;48(3):106455. doi: 10.1016/j.htct.2026.106455 (PMC13092737; doi:10.1016/j.htct.2026.106455)
Supplement: Supplementary file 2 [file mmc2.docx]

**Supplementary Table 1:** Characteristics of acute myeloid leukemia patients from the Beat AML^1^ study and their association with the 3-gene *TP53* mutant-like score

| **Factor** | **Total** | **3-gene *TP53* mutant-like score** | | |  |  |  |  |
| --- | --- | --- | --- | --- | --- | --- | --- | --- |
|  |  | **Low** | **Intermediate** | **High** | ***p***^2^******* | ***p***^2^******** | ***p***^2^********* | ***p***^2^********** |
| **AML patient** | **197** | **44** | **87** | **66** |  |  |  |  |
| Gender - n  Male/Female | 97/100 | 17/27 | 48/39 | 32/34 | 0.33 | 0.10 | 0.42 | 0.20 |
| Age (years) - median (range): | 52 (2 - 85) | 47 (2 - 84) | 50.5 (8 - 84) | 60 (8 - 85) | **0.01** | 0.45 | **0.02** | **0.02** |
| Bone marrow blasts (%) - median (range) | 80 (1.5 -98) | 71 (7 - 97) | 75 (14 - 98) | 84(1.5 - 97) | **<0.0001** | 0.17 | **0.006** | **0.001** |
| White blood cell count × 10^9^/L | 38.83 (1.5 – 427.46) | 30.60 (3.6 - 117.53) | 31.9 (1.5 - 427.46) | 60.18 (2.41 - 183.49) | **0.004** | 0.42 | **0.02** | **0.009** |
| ELN 2022 - n |  |  |  |  | **0.003** | **0.006** | 0.17 | **0.003** |
| Favorable | 104 | 33 | 40 | 31 |  |  |  |  |
| Intermediate | 65 | 6 | 30 | 29 |  |  |  |  |
| Adverse | 28 | 5 | 17 | 6 |  |  |  |  |

TCGA: The Cancer Genome Atlas; AML: acute myeloid leukemia; ELN: European LeukemiaNet

^1^The clinical and laboratorial data of TCGA AML cohort were obtained from the cBioPortal for Cancer Genomics (http://www.cbioportal.org)

^2^For statistical analyses, Mann-Whitney test or Kruskal-Wallis test was used for continuous variables, and Fisher's exact test or Chi-squared test was used for categorical factors.

* High versus Low.

** Intermediate versus Low.

*** High versus Intermediate.

**** High versus Intermediate *versus* Low.

| **Supplementary Table 2.** Acute myeloid leukemia patients’ characteristics from TCGA AML^1^ study and their association with 3-gene *TP53* mutant-like score | | | | | | | | |  |
| --- | --- | --- | --- | --- | --- | --- | --- | --- | --- |
| **Factors** | **Total** | **3-gene *TP53* mutant-like score** | | |  |  |  |  | |
|  |  | **Low** | **Intermediate** | **High** | ***p***^3^******* | ***p***^3^******** | ***p***^3^********* | ***p***^3^********** |  |
| **AML patients** | **113** | **53** | **38** | **22** |  |  |  |  |  |
| Sex  Male/Female | 59/54 | 26/27 | 24/14 | 9/13 | 0.06 | 0.21 | 0.11 | 0.21 |  |
| Age (years), median (range): | 54 (18 - 81) | 51 (21-72) | 57 (18-77) | 55.5 (21-81) | 0.48 | 0.09 | 0.61 | 0.25 |  |
| Bone marrow blasts (%), median (range) | 74 (34 -100) | 64 (34-95) | 79.5 (43-100) | 83 (39-99) | **0.002** | **0.004** | 0.32 | **0.001** |  |
| White blood cell count × 10^9^/L | 30.9 (0.6 – 297.4) | 18.7(0.6-101.3) | 37.3 (1.2-171.9) | 82.1 (2.3-297.4) | **<0.001** | **0.02** | **0.04** | **<0.001** |  |
| Cytogenetic risk^2^ |  |  |  |  | **0.03** | 0.53 | 0.14 | 0.13 |  |
| Good | 17 | 12 | 5 | 0 |  |  |  |  |  |
| Intermediate | 78 | 32 | 27 | 19 |  |  |  |  |  |
| Poor | 16 | 8 | 5 | 3 |  |  |  |  |  |
| N.A. | 2 | 1 | 1 | 0 |  |  |  |  |  |
| Molecular risk^2^ |  |  |  |  | **0.03** | 0.21 | 0.12 | 0.07 |  |
| Good | 17 | 12 | 5 | 0 |  |  |  |  |  |
| Intermediate | 72 | 28 | 27 | 17 |  |  |  |  |  |
| Poor | 22 | 12 | 5 | 5 |  |  |  |  |  |
| N.A. | 2 | 1 | 1 | 0 |  |  |  |  |  |

Abbreviations: TCGA, The Cancer Genome Atlas; AML, acute myeloid leukemia; N.A., not available.

^1^The clinical and laboratorial data of TCGA AML cohort were obtained from cBioPortal for Cancer Genomics (http://www.cbioportal.org).

^2^Cytogenetic risk and Molecular risk was stratified according TCGA study; 2 AML patients were not classified.

^3^For statistical analyzes, Mann–Whitney test or Kruskal-Wallis test was used for measured factors, and Fisher's exact test or Chi-squared test was used for categorical factors.

* High *versus* Low.

** Intermediate *versus* Low.

*** High *versus* Intermediate.

**** High *versus* Intermediate *versus* Low.
