# Supplementary material for: Exploring the prognostic impact and biological functions of mutant-like TP53-related genes in acute myeloid leukemia
Source: Hematol Transfus Cell Ther. 2026 Apr 14;48(3):106455. doi: 10.1016/j.htct.2026.106455 (PMC13092737; doi:10.1016/j.htct.2026.106455)
Supplement: Supplementary file 3 [file mmc3.docx]

| **Supplementary Table 3.** Univariate and multivariate analysis for overall survival of acute myeloid leukemia patients from Beat AML cohort according to clinical and laboratorial characteristics and 3-gene *TP53* mutant-like score. | | | | | | |
| --- | --- | --- | --- | --- | --- | --- |
| **Factors** | **Overall survival** | | | | | |
|  | **Univariate** | | | **Multivariate** | | |
|  | **HR**^1^ | **(95% C.I.)** | ***p***^3^ | **HR**^1^ | **(95% C.I.)** | ***p***^3^ |
| **Sex**  Male *vs* female | 0.73 | 0.47 – 1.14 | 0.17 | 0.67 | 0.42 – 1.08 | 0.10 |
| **Diagnosis age**^2^ | 1.03 | 1.02 – 1.05 | **<0.0001** | 1.03 | 1.02 – 1.05 | **<0.0001** |
| **White blood cell count**^2^ | 1.00 | 0.99 – 1.004 | 0.75 | 0.999 | 0.99 – 1.003 | 0.66 |
| **ENL 2022^3^**  Favorable *vs.* intermediate *vs.* adverse | 1.16 | 0.87 – 1.55 | 0.32 | 1.23 | 0.90 – 1.69 | 0.19 |
| **3-gene TP53 mutant-like score^3^**  High *vs.* intermediate *vs.* low | 1.91 | 1.38 – 2.66 | **<0.0001** | 1.48 | 1.03 – 2.13 | **0.03** |

Abbreviations: TCGA, The Cancer Genome Atlas.

Significant statistical differences are highlighted in bold.

^1^Hazard ratios (HR)> 1 indicates that the increase in values for continuous variable or the first factor for categorical variable has a worse outcome.

^2^Factors were analyzed as continuous variables.

^3^Absent values were excluded in the calculation of the *p* values.
