# Supplementary material for: Exploring the prognostic impact and biological functions of mutant-like TP53-related genes in acute myeloid leukemia
Source: Hematol Transfus Cell Ther. 2026 Apr 14;48(3):106455. doi: 10.1016/j.htct.2026.106455 (PMC13092737; doi:10.1016/j.htct.2026.106455)
Supplement: Supplementary file 4 [file mmc4.docx]

| **Supplementary Table 4.** Univariate and multivariate analysis for overall survival of acute myeloid leukemia patients from TCGA cohort according to clinical and laboratorial characteristics and 3-gene *TP53* mutant-like score. | | | | | | |
| --- | --- | --- | --- | --- | --- | --- |
| **Factors** | **Overall survival** | | | | | |
|  | **Univariate** | | | **Multivariate** | | |
|  | **HR**^1^ | **(95% C.I.)** | ***p***^3^ | **HR**^1^ | **(95% C.I.)** | ***p***^3^ |
| **Sex**  Male *vs* female | 1.06 | 0.66 – 1.71 | 0.80 | 1.11 | 0.69 – 1.81 | 0.66 |
| **Diagnosis age**^2^ | 1.01 | 0.99 – 1.03 | 0.17 | 1.01 | 0.99 – 1.03 | 0.14 |
| **White blood cell count**^2^ | 1.01 | 1.00 – 1.01 | **0.001** | 1.004 | 0.99 – 1.01 | 0.10 |
| **Molecular risk^3^**  Poor *vs.* intermediate *vs.* good | 1.50 | 1.01 – 2.23 | **0.04** | 1.67 | 1.06 – 2.62 | **0.03** |
| **3-gene TP53 mutant-like score^3^**  High *vs.* intermediate *vs.* low | 1.81 | 1.36 – 2.43 | **<0.0001** | 1.57 | 1.13 – 2.20 | **0.007** |

Abbreviations: TCGA, The Cancer Genome Atlas.

Significant statistical differences are highlighted in bold.

^1^Hazard ratios (HR)> 1 indicates that the increase in values for continuous variable or the first factor for categorical variable has a worse outcome.

^2^Factors were analyzed as continuous variables.

^3^Absent values were excluded in the calculation of the *p* values.
